# Risk stratification of tuberculosis transmission in healthcare settings: A systematic review

**DOI:** 10.1017/ash.2025.386

**Published:** 2025-09-24

**Authors:** Fizza Manzoor, Chanu Rhee, Meghan Baker, Michael Klompas

**Affiliations:** 1 Brigham and Women’s Hospital; 2 Brigham and Women’s Hospital/Harvard Medical School; 3 Harvard Medical School

## Abstract

**Background:** Tuberculosis transmission in healthcare is poorly understood. Exposure definitions for patients and healthcare workers tend to be based on custom rather than data leading to many people being flagged for evaluation despite few infection transmission events. We reviewed the medical literature to identify and quantify risk factors for tuberculosis transmission in healthcare to guide risk-stratification and inform exposure definitions. **Methods:** We reviewed MEDLINE, EMBASE, CINAHL and Cochrane databases from inception to December 10, 2024. We included studies reporting tuberculosis transmission from infected adult patients to healthcare workers and other patients in both inpatient and outpatient settings. We evaluated 12 transmission risk factors: contact factors (exposure duration, proximity of exposure, mask use, room ventilation), patient factors (smear positivity, NAAT positivity, cavitary pulmonary disease, respiratory symptoms), and procedure factors (intubation, bronchoscopy, sputum collection, and other procedures). **Results:** A total of 6,695 studies were identified of which 49 met inclusion criteria. Contact factors associated with increased risk of transmission included poor room ventilation (≤ 2 air exchanges per hour, 60-70% air recirculation without high efficiency filtration, high ambient carbon dioxide levels with median 660-800 parts per million) and positive pressure air flow from poorly ventilated rooms to nearby clinical spaces. Most ventilation-related transmissions occurred before modern healthcare ventilation standards were implemented. Sustained proximity to infected patients was associated with patient-to-patient transmission via shared rooms (4 transmissions/90 exposures, minimum exposure ≥16 hours) and prolonged residence adjacent to a poorly ventilated standard pressure room (42 transmissions/430 exposures, minimum exposure ≥24 hours). Amongst 766 cases of tuberculosis transmission from patients to healthcare workers, risk factors included prolonged patient contact (median 6 hours, minimum 30 minutes), failure to wear an N95 respirator, and face-to-face patient care (28% of transmissions were associated with face-to-face patient contact, 21% of transmissions were associated with working on the same unit without direct patient contact, and contact details were unknown in 52% of transmissions). Patient factors associated with increased transmission included cavitary disease (OR 1.90, 95% CI 1.26-2.84). Transmission risk was similar for smear-positive and smear-negative patients undergoing aerosol-generating procedures without airborne precautions (17/111 smear negative exposures led to transmission vs 32/166 smear positive exposures, 15% vs 19%). All transmissions to healthcare workers associated with intubation, bronchoscopy and induced sputum collection occurred without airborne precautions. **Conclusions:** Detailed review of the circumstances around nosocomial tuberculosis exposure helps identify transmission risk factors that can inform more evidence-based, detailed and individualized exposure definitions.

